# Diversification of the rainfrog *Pristimantis ornatissimus* in the lowlands and Andean foothills of Ecuador

**DOI:** 10.1371/journal.pone.0172615

**Published:** 2017-03-22

**Authors:** Juan M. Guayasamin, Carl R. Hutter, Elicio E. Tapia, Jaime Culebras, Nicolás Peñafiel, R. Alexander Pyron, Carlos Morochz, W. Chris Funk, Alejandro Arteaga

**Affiliations:** 1 Universidad San Francisco de Quito USFQ, Colegio de Ciencias Biológicas y Ambientales COCIBA, Instituto de Investigaciones Biológicas y Ambientales BIÓSFERA, Laboratorio de Biología Evolutiva, Campus Cumbayá, Quito, Ecuador; 2 Centro de Investigación de la Biodiversidad y Cambio Climático (BioCamb), Ingeniería en Biodiversidad y Cambio Climático, Facultad de Medio Ambiente, Universidad Tecnológica Indoamérica, Calle Machala y Sabanilla, Quito, Ecuador; 3 Department of Ecology and Evolutionary Biology, Biodiversity Institute, University of Kansas, Lawrence, Kansas, United States of America; 4 Centro Jambatu de Investigación y Conservación de Anfibios, Fundación Otonga, San Rafael, Quito, Ecuador; 5 Department of Biological Sciences, The George Washington University, Washington, D.C., United States of America; 6 Reserva de Biodiversidad Mashpi, Quito, Ecuador; 7 Department of Biology, Graduate Degree Program in Ecology, Colorado State University, Fort Collins, Colorado, United States of America; 8 Tropical Herping, Torres Santa Fe, Quito, Ecuador; National Cheng Kung University, TAIWAN

## Abstract

Geographic barriers and elevational gradients have long been recognized as important in species diversification. Here, we illustrate an example where both mechanisms have shaped the genetic structure of the Neotropical rainfrog, *Pristimantis ornatissimus*, which has also resulted in speciation. This species was thought to be a single evolutionary lineage distributed throughout the Ecuadorian Chocó and the adjacent foothills of the Andes. Based on recent sampling of *P*. *ornatissimus* sensu lato, we provide molecular and morphological evidence that support the validity of a new species, which we name *Pristimantis ecuadorensis* sp. nov. The sister species are elevational replacements of each other; the distribution of *Pristimantis ornatissimus* sensu stricto is limited to the Ecuadorian Chocó ecoregion (< 1100 m), whereas the new species has only been found at Andean localities between 1450–1480 m. Given the results of the Multiple Matrix Regression with Randomization analysis, the genetic difference between *P*. *ecuadorensis* and *P*. *ornatissimus* is not explained by geographic distance nor environment, although environmental variables at a finer scale need to be tested. Therefore this speciation event might be the byproduct of stochastic historic extinction of connected populations or biogeographic events caused by barriers to dispersal such as rivers. Within *P*. *ornatissimus* sensu stricto, morphological patterns and genetic structure seem to be related to geographic isolation (e.g., rivers). Finally, we provide an updated phylogeny for the genus, including the new species, as well as other Ecuadorian *Pristimantis*.

## Introduction

Species diversification is important to understanding the great diversity of organisms in nature. The mechanisms by which this diversity has arisen has been a long-standing question since the seminal works of Darwin and Wallace [1,2,3]. There are numerous hypotheses to explain the factors involved in speciation, and for the majority of species, the most likely scenario includes the geographic separation of an ancestral population into two or more lineages, resulting in allopatric speciation [4,5,6]. The resulting lineages may [7,8,9,10,11] or may not [12,13,14] maintain their ancestral ecological requirements.

When sister species occur parapatrically along elevational gradients, speciation has been hypothesized to include the following steps: (1) an ancestral population colonizes a new environment, (2) gene flow becomes restricted between populations, and (3) separate lineages evolve and are maintained [15]. Alternatively, ecological speciation may occur across an environmental gradient, in which divergent ecological selection results in reproductive isolation, for example through pleiotropy or close genetic linkage between traits involved in local adaptation and mate choice [16,17].

Studying closely related populations that have relatively restricted, but complex geographic distributions can shed light on the evolution of population genetic structure and the origination of species [18]. Our study focuses on one of the most distinctive anuran species endemic to Ecuador, *Pristimantis ornatissimus* Despax, 1911 [19]. This taxon has a striking yellow-and-black dorsal pattern that is unique in the genus [15,20]. Recent fieldwork has revealed differences in color pattern among populations of *P*. *ornatissimus* sensu lato. Further morphological and molecular (mtDNA) analyses indicate that highland populations represent a new species that we describe herein, and that variation observed among lowland populations seem to be the result of different degrees of geographic isolation (for example, caused by the Guayllabamba-Esmeraldas river). This study describes how a lineage diversifies, simultaneously, through different mechanisms (barriers, elevational gradients), a pattern that might be shared by other species with low vagility.

## Methods

### Ethics statement

This study was carried out in strict accordance with the guidelines for use of live amphibians and reptiles in field research compiled by the American Society of Ichthyologists and Herpetologists (ASIH), The Herpetologists' League (HL) and the Society for the Study of Amphibians and Reptiles (SSAR). All procedures with animals were approved by the Centro de Investigación de la Biodiversidad y Cambio Climático (BioCamb), Universidad Tecnológica Indoamérica. Additionally, procedures were reviewed and approved by the Ministerio de Ambiente del Ecuador (MAE). Research permits were issued by the Ministerio de Ambiente del Ecuador (MAE-DNB-CM-2015-0017, granted to Universidad Tecnológica Indoamérica; and N°005–15 IC-FAU-DNB/MA, granted to Centro Jambatu de Investigación y Conservación de Anfibios).

### Nomenclature

We use the genus *Pristimantis* Jiménez de la Espada, 1870 [21], as defined by Heinicke *et al*. [22]. For a discussion on the systematics of Terrarana, see Padial *et al*. [23]. For a current list of the species in the genus *Pristimantis*, see Frost [24] and AmphibiaWeb [25].

### Nomenclatural acts

The electronic edition of this article conforms to the requirements of the amended International Code of Zoological Nomenclature, and hence the new names contained herein are available under that Code from the electronic edition of this article. This published work and the nomenclatural acts it contains have been registered in ZooBank, the online registration system for the ICZN. The ZooBank LSIDs (Life Science Identifiers) can be resolved and the associated information viewed through any standard web browser by appending the LSID to the prefix “http://zoobank.org/”. The LSID for this publication is: urn:lsid:zoobank.org:pub:A1586F9A-8C91-417E-A0E1-47898A9086CF. The electronic edition of this work was published in a journal with an ISSN, and has been archived and is available from the following digital repositories: PubMed Central and LOCKSS.

### Morphology

We examined alcohol-preserved specimens from the herpetology collection at the Museo de Zoología of the Universidad Tecnológica Indoamérica, Quito, Ecuador (MZUTI), Centro Jambatu de Investigación y Conservación de Anfibios, San Rafael, Ecuador (CJ), Museum d'Histoire Naturelle, Geneva, Switzerland (MHNG), and University of Kansas Museum of Natural History, Division of Herpetology, Lawrence, Kansas, USA (KU). To facilitate comparison, the description and diagnosis of the new species follows the format of Lynch and Duellman [15]. Fingers are numbered from I–IV. Morphological measurements were taken with Mitutoyo^®^ digital caliper to the nearest 0.1 mm, as described by Guayasamin [26]: (1) snout–vent length (SVL), (2) tibia length, (3) foot length, (4) head length, (5) head width, (6) eye-to-nostril distance, (7) tympanum diameter, (8) radioulna length, and (9) hand length. Sexual maturity was determined by the presence of vocal slits in males and by the presence of eggs or convoluted oviducts in females. Color patterns were compared using preserved specimens and photographic records. Examined specimens are listed in Table 1 or are part of the type series of the new species described herein.

**Table 1:** Gene sampling of *Pristimantis ornatissimus* and *P*. *ecuadorensis*, with their corresponding Genbank numbers.

Museum number	Locality (Province: locality)	Elevation (m)	Latitude	Longitude	Genbank accession number		
					12S	16S	ND1
***Pristimantis ornatissimus* sensu stricto**							
MZUTI 3509	Pichincha: Milpe	992	0,03884	-78,87092	KU574595	KU574609	KU720472
MZUTI 3749	Pichincha: Milpe	1044	0,03905	-78,87054	KU574596	KU574610	KU720471
MZUTI 4106	Pichincha: Reserva de Biodiversidad Mashpi	901	0,16497	-78,87736	KU574594	KU574611	KU720473
MZUTI 4326	Pichincha: Reserva de Biodiversidad Mashpi	1100	0,16414	-78,86944	—	KU574612	KU720474
MZUTI 4329	Pichincha: Reserva de Biodiversidad Mashpi	950	0, 16594	-78,87837	—	KU574613	KU720475
MZUTI 4798	Esmeraldas: Reserva Canandé	310	0,5258	-78,2088	KU720464	KU720463	KU720480
CJ 4039	Esmeraldas: Parroquia Alto Tambo: Reserva Otokiki	638	0,91325	-78,568	KU574593	KU574604	---
CJ 4087	Esmeraldas: Parroquia Alto Tambo: Reserva Otokiki	638	0,91325	-78,568	KU574588	KU574605	KU720476
CJ 4088	Esmeraldas: Parroquia Alto Tambo: Reserva Otokiki	638	0,91325	-78,568	KU574589	KU574606	KU720478
CJ 4089	Esmeraldas: Parroquia Alto Tambo: Reserva Otokiki	638	0,91325	-78,568	KU574590	KU574607	KU720477
CJ 4090	Esmeraldas: Parroquia Alto Tambo: Reserva Otokiki	638	0,91325	-78,568	KU574591	KU574608	KU720479
CJ 4085	Santo Domingo de los Tsáchilas: Sapo Parque La Florida, ca. 4 km W of La Florida.	884	-0.253	-79.030	KU574587	KU574602	KU720469
CJ 4092	Santo Domingo de los Tsáchilas: Sapo Parque La Florida, ca. 4 km W of La Florida.	884	-0.253	-79.030	KU574592	KU574603	KU720470
MZUTI 4806	Cotopaxi. Reserva El Jardín de los Sueños, near La Maná,	349	-0.831	-79.21	KX785337	KX785341	KX785345
MZUTI 4807	Cotopaxi. Reserva El Jardín de los Sueños, near La Maná,	349	-0.831	-79.21	KX785338	KX785342	KX785346
***Pristimantis ecuadorensis* sp. nov.**							
CJ 4060	Cotopaxi: Farm owned by César Tapia, ca. 3 km NE of San Francisco de Las Pampas	1480	-0,42415	-78,9572	KU574597	KU574598	KU720465
CJ 4082	Cotopaxi: Farm owned by César Tapia, ca. 3 km NE of San Francisco de Las Pampas	1480	-0,42415	-78,9572	KU574584	KU574599	KU720466
CJ 4083	Cotopaxi: Farm owned by César Tapia, ca. 3 km NE of San Francisco de Las Pampas	1480	-0,42415	-78,9572	KU574585	KU574600	KU720468
CJ 4084 (holotype)	Cotopaxi: Farm owned by César Tapia, ca. 3 km NE of San Francisco de Las Pampas	1480	-0,42415	-78,9572	KU574586	KU574601	KU720467
CJ 5350	Cotopaxi: Farm owned by César Tapia, ca. 3 km NE of San Francisco de Las Pampas	1480	-0,42415	-78,9572	KX785339	KX785343	KX785347
CJ 5351	Cotopaxi: Farm owned by César Tapia, ca. 3 km NE of San Francisco de Las Pampas	1480	-0,42415	-78,9572	KX785340	KX785344	KX785348

### Molecular data

#### DNA extraction, amplification, and sequencing

Genomic DNA was extracted from ethanol-preserved tissue with a salt precipitation method using the Puregene DNA purification kit (Gentra Systems). We amplified and sequenced the mitochondrial 12S rRNA, 16S rRNA and NADH dehydrogenase subunit I (ND1) gene regions. A portion of the 12S marker was obtained using the primers 12L29E-F (AAAGCRTAGCACTGAAAATGCTAAGA) and 12H46E-R (GCTGCACYTTGACCTGACGT) developed by Heinicke *et al*. [22]. The 16S gene was amplified partially with the primers 16SC (GTRGGCCTAAAAGCAGCCAC) and 16Sbr-H (CCGGTCTGAACTCAGATCACGT) developed by Darst and Cannatella [27] and Palumbi *et al*. [28], respectively. The complete sequence of gene ND1 and adjacent 16S and tRNA genes was retrieved with the use of primers ND1-16S-frog-F (TTACCCTRGGGATAACAGCGCAA) and ND1-tMet-frog-R (TTGGGGTATGGGCCCAAAAGCT) [29]. Each PCR reaction contained a final concentration of 3 mM MgCl_2_, 0.2 mM dNTPs, 0.05 U/μL *Taq* DNA polymerase (Invitrogen) and 0.2 μM each primer, in a total volume of 25 μL. DNA amplification included a 3-min initial denaturation step at 94°C and a final extension of 7 min at 72°C. For 12S, 9 cycles of 30 sec at 93°C, 30 sec at 60°C decreasing 1°C/cycle, 1 min at 72°C, followed by 15–25 cycles (depending on initial DNA template amount) of 30 sec at 93°C, 30 sec at 52°C, 1 min at 72°C were performed. For 16S amplification, PCR conditions were the same as those for 12S, except that touchdown consisted in 10 cycles ranging from 67 to 58°C, followed by 18 or 20 cycles at 58°C. PCR parameters for ND1 consisted of 22 cycles of 30 sec at 93°C, 45 sec at 66°C, and 1 min at 72°C. Single PCR products were visualized in 1.5% agarose gel, and unincorporated primers and dNTPs were removed from PCR products with illustra ExoStar enzymes (GE Healthcare Life Sciences). Sequencing was conducted at Macrogen Inc. (South Korea).

### Genetic differentiation within *Pristimantis ornatissimus* sensu lato

To determine the relationships and genetic distances among populations of *Pristimantis ornatissimus* sensu lato, we sampled a total of 21 individuals representing seven populations, covering an elevational gradient from 310 to 1480 m. The sequences from the three mitochondrial genes (12S, 16S, ND1) were independently aligned using MAFFT v7 [30], with the Q-INS-i strategy. Maximum likelihood (ML) trees and branch lengths were estimated using GARLI 2.01 (Genetic Algorithm for Rapid Likelihood Inference [31]) for each gene. Mitochondrial genes were analyzed separately because population sampling differed among genes (Table 1) and we aimed to assess congruence in genetic patterns. GARLI uses a genetic algorithm that finds the tree topology, branch lengths, and model parameters that maximize lnL simultaneously [31]. Individual solutions were selected after 10,000 generations with no significant improvement in likelihood, with the significant topological improvement level set at 0.01; the final solution was selected when the total improvement in likelihood score was lower than 0.05, compared to the last solution obtained. Default values were used for other GARLI settings, as per recommendations of the developer [30]. Bootstrap support was assessed via 1000 pseudoreplicates under the same settings used in tree search. GenBank accession numbers are listed in Table 1.

### *Pristimantis* phylogenetic relationships

To determine the phylogenetic position of *Pristimantis ornatissimus* and the new species, we aligned sequences generated during this study (Table 1) with corresponding mitochondrial markers from all other available species (245 terminals) of *Pristimantis* from GenBank (http://www.ncbi.nlm.nih.gov/genbank). As outgroups we included 53 species from Craugastoridae as well as 28 species from other families in Hyloidea (S1 Appendix). All matrices were aligned as described above. Our final mtDNA matrix included 347 species spanning 4,358bp for phylogenetic analysis.

For maximum likelihood (ML) estimation of phylogenetic relationships, we follow the phylogenetic methods described in the previous section. For Bayesian Inference analyses, we used MrBayes 3.2.4 [32]. We first used JModelTest 2.0 [33] to select the model of sequence evolution that best fits the data, using the Bayesian information criterion (BIC; [34]). We did not evaluate models that include both gamma-distributed rate variation across sites (G) and a proportion of invariant sites (I) because a correlation between parameters can affect their joint estimation [35]. The best-fit model for the 16S and 12S stem partitions was SYM + I + G model (Symmetrical Model with equal base frequencies, proportion of invariant sites, and a gamma distribution for 45 rates across sites). The best-fit model for the 12S, 16S loop partitions as well as ND1 was the GTR + Γ model (Generalized Time-Reversible + Gamma; accounting for rate heterogeneity and variable base frequencies across sites to approximate a gamma-shaped distribution).

Finally, we conducted an analysis for 100 million generations (sampling every 10,000) with four Markov chains and default heating values. We ran the analyses at least twice to assess consistent convergence and stationarity, where we examined the standard deviation of split frequencies and plotted the –lnL per generation. We discarded the trees generated before stationarity as ‘burn-in’, which was the first 25% of trees.

### Isolation by distance or by environment

Genetic differences among populations of *Pristimantis ornatissimus* sensu lato could be explained by at least the following hypotheses: (1) Isolation by Distance (IBD), where patterns of genetic differentiation are explained solely by geographic distance, or (2) Isolation by Environment (IBE), where genetic differentiation is explained by environmental isolation [36]. To support IBD, the genetic divergence among *Pristimantis ornatissimus* sensu lato populations would be explained by geographic distance. In contrast, if a population represents a distinct species, then it should continue to be genetically distinct from all other populations after controlling for geographic distance. To support IBE, the genetic divergence among *P*. *ornatissimus* sensu lato populations would be correlated with environmental dissimilarity, suggesting a role of adaptive divergence in response to differing environmental pressures in causing speciation. To test these hypotheses, we used uncorrected p distances calculated from concatenated 12S rRNA, 16S rRNA, and ND1 sequences, using only individuals with data from all three genes, as our measure of genetic distance among individuals.

Since recent studies suggest that Mantel tests generate biases (i.e., low p-values) in systems with autocorrelated variables [37], we used Multiple Matrix Regression with Randomization (MMRR) to estimate the independent effects on genetic differentiation from geographic distance and environmental dissimilarity among the seven populations, as well as excluding the population from Las Pampas [38]. We used the WorldClim dataset and extracted data from 19 climatic variables for each of the seven study sites, at 30 second resolution [39]. Next, we used a principal component analysis (PCA) on the 19 BioClim variables to convert these potentially correlated variables into uncorrelated ones. We selected the first three principal components, which represented 87.2% of the variation in the dataset (PC loadings and alternative results using a subset of uncorrelated BioClim variables can be found in S1 Table). Temperature and precipitation variables were strongly associated with these axes. We calculated environmental dissimilarity matrices of the different study sites using Euclidean distances among the three PC axes. To estimate geographic distance, we calculated a Euclidean distance matrix in kilometers among each of the study sites. Finally, we used the MMRR code provided in Wang [38] with the genetic, geographic, and environmental distance matrices for 10,000 permutations. For this analysis we used the following R statistical software packages: RASTER [40], DISMO [41], and APE [42].

## Results

### Color differentiation within *Pristimantis ornatissimus* sensu lato

**Fig 1:**
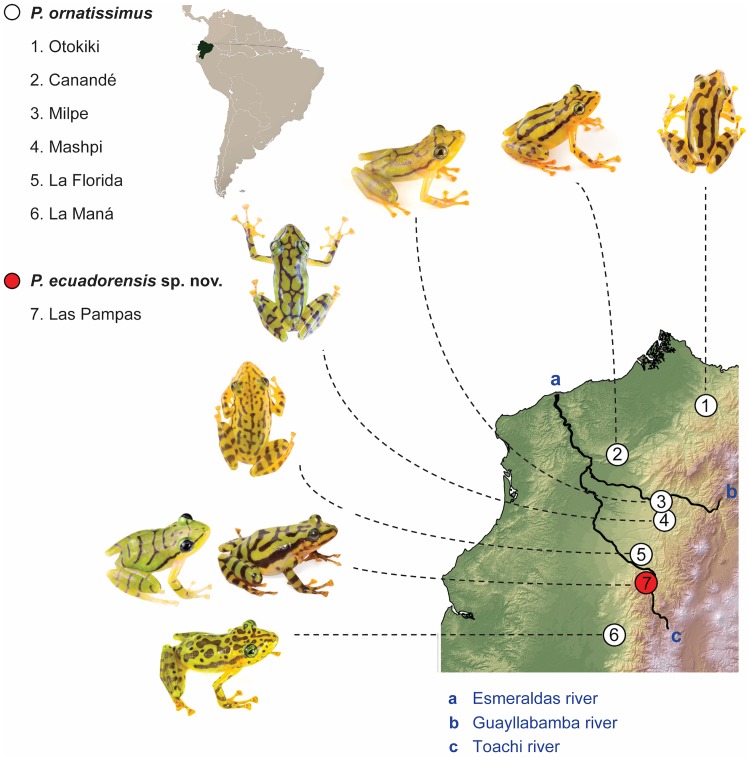
Color variation in sequenced *Pristimantis ornatissimus* sensu stricto and *P*. *ecuadorensis* sp. nov. in Ecuador.

**Fig 2:**
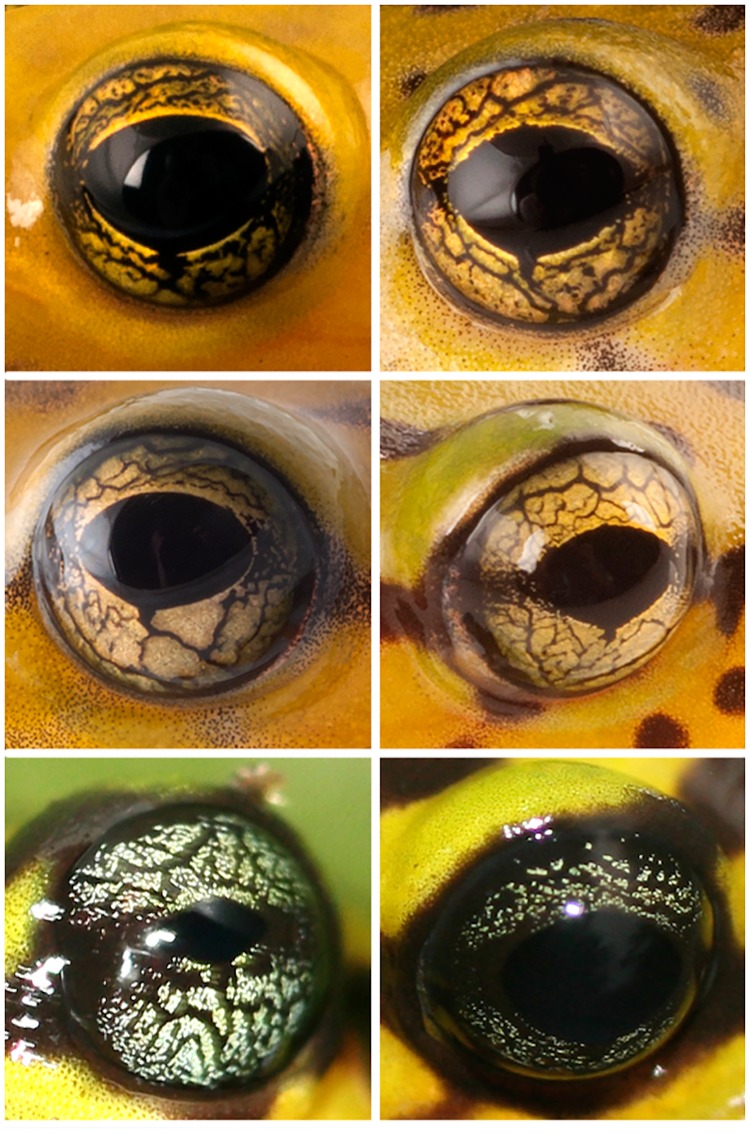
Variation in iris coloration in life of *Pristimantis ornatissimus* sensu stricto and *P*. *ecuadorensis* sp. nov. First (CJ 4092, CJ 4085) and second (no voucher, CJ 4087) rows show iris color of *Pristimantis ornatissmus* sensu stricto. Bottom row illustrates coloration of *P*. *ecuadorensis* sp. nov (no voucher, CJ 4082).

**Fig 3:**
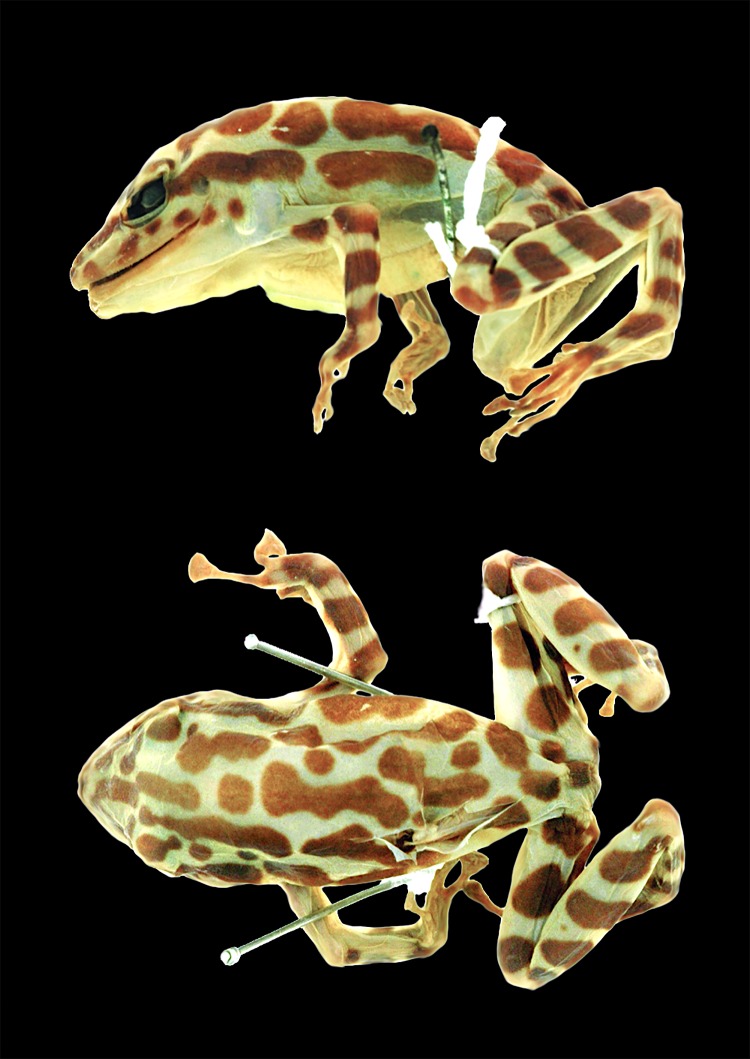
Holotype of *Pristimantis ornatissimus*, MNHNP 1906.264. Note matching color patter with populations from near La Maná (Fig 1).

The observed variation in dorsal and iris coloration and patterns in *Pristimantis ornatissimus* sensu lato is shown in Figs 1 and 2. Patterns are not random and can be associated to specific geographic localities, as follows:

- *Lowlands (north)*: Populations of *P*. *ornatissimus* north of Guayllabamba-Esmeraldas river. Dorsal pattern with continuous longitudinal black stripes, lacking spots (Fig 1). Iris yellow to gold with thin black reticulations (Fig 2). Examined specimens: MZUTI 4798 (Reserva Canandé), CJ 4039, 4087–90, 4089 (Reserva Otokiki),
- *Lowlands (center)*: Populations of *P*. *ornatissimus* south of Guayllabamba-Esmeraldas river and north of Toachi river. Reticulated dorsal color pattern, usually lacking spots (some individuals from La Florida and Santo Domingo de los Tsáchilas have spots). Iris yellow to gold with thin black reticulations (Fig 2). Examined specimens: MZUTI 3749, 3509 (Milpe), 4326, 4329, 4799, 4800, 4106 (Mashpi), 119744–47 (Santo Domingo de los Tsáchilas), CJ 4085, 4092, 4758 (La Florida).
- *Lowlands (south)*: Populations south of Toachi river. Dorsal color pattern with discontinuous longitudinal stripes and blotches and/or spots (Fig 1). Iris yellow to gold with thin black reticulations (Fig 2). Examined specimens: MNHNP 1906.264 (holotype; Fig 3), MZUTI 4806, 4807, KU 141970–71 (La Maná), KU 141967–68 (Río Baba).
- *Highlands*: Populations found above 1400 m. Dorsum with transverse black stripes that often times form a reticulated pattern. Iris varies from light blue to grayish green or grayish yellow (Fig 2). We recognize the highland populations as a new species, which we describe below. Examined specimens: KU 221684 (Palo Quemado), CJ 4060, 4082, 4084, 5350, 5351, MHNG 2251.090, 2251.091, 2271.097, 2271.098, 2313.083, 2313.084, 2251.061–65, 2571.075 (San Francisco de Las Pampas).

### Genetic differentiation within *Pristimantis ornatissimus* sensu lato

**Fig 4:**
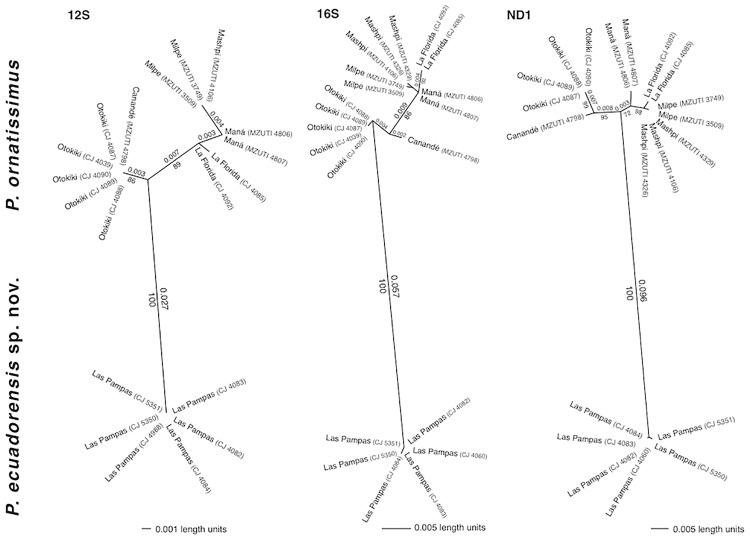
Mitochondrial genetic structure among populations of *Pristimantis ornatissimus* and *P*. *ecuadorensis* sp. nov. as inferred from maximum likelihood analyses. Branch lengths and significant bootstrap values are presented for each tree. Note reciprocal monophyly and genetic distances between *P*. *ornatissimus* sensu stricto and *P*. *ecuadorensis* sp. nov.

We generated a total of 60 mitochondrial sequences for 21 specimens (Table 1). Independent analyses of the three genes (Fig 4) show that the species currently recognized as *Pristimantis ornatissimus* is composed of two clearly differentiated lineages, one from lowland localities (< 1100 m: Reserva Otokiki, Reserva Canandé, Milpe, Reserva de Biodiversidad Mashpi, La Florida, La Maná), which corresponds to *P*. *ornatissimus* Despax, 1991, sensu stricto, and the other from highlands (> 1400 m: Las Pampas) that we describe as a new species below. Lowland and highland lineages are reciprocally monophyletic (Fig 4). The average uncorrected genetic distance between lowland and highland individuals is 2.7% (12S), 5.7% (16S), and 9.6% (ND1; Fig 4). Additionally, genetic structure is evident among populations of the lowland species (*P*. *ornatissimus* sensu stricto), but genetic distances are always below 1%.

### Isolation by distance or by environment

**Fig 5:**
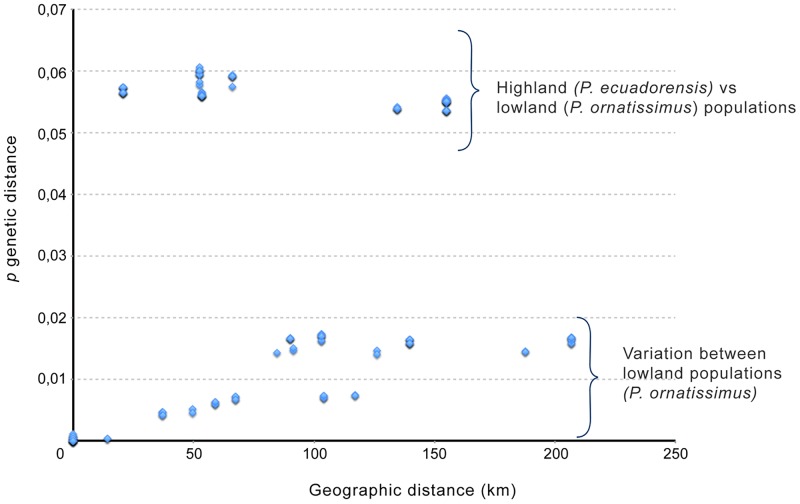
Isolation-by-distance among sequenced individuals. Note than populations from highlands (*P*. *ecuadorensis*) are much more divergent from *P*. *ornatissimus* sensu stricto than expected based on geographic distance (see Results).

The results of the MMRR analyses show that geographic and environmental dissimilarity—measured using the first three axes from the PCA of 19 Bioclim variables—do not explain genetic differentiation among populations (Table 2; Fig 5). When MMRR analyses were performed excluding populations from Las Pampas, similar results were obtained. An alternative analysis that selected uncorrelated raw climatic variables was also not significant and was therefore consistent with the PCA results (see S2 Table for details).

**Table 2:** Results of the Multiple Matrix Regression with Randomization (MMRR) analyses, which tests whether geographic and environmental dissimilarity influence genetic differentiation among populations, were not significant. PC axes were obtained from a PCA analysis of 19 Bioclim variables [39]. Variables were ordered by significance in the model (T P-value).

Variable	Coefficient	T statistic	T P-value	F statistic	F P-value
Intercept	0.03	7.303	0.761		
Geographic	0	-0.505	0.543		
PC1	0	0.434	0.675		
PC2	0	0.203	0.855		
PC3	0.002	0.998	0.35	0.472	0.826

### *Pristimantis* relationships

**Fig 6:**
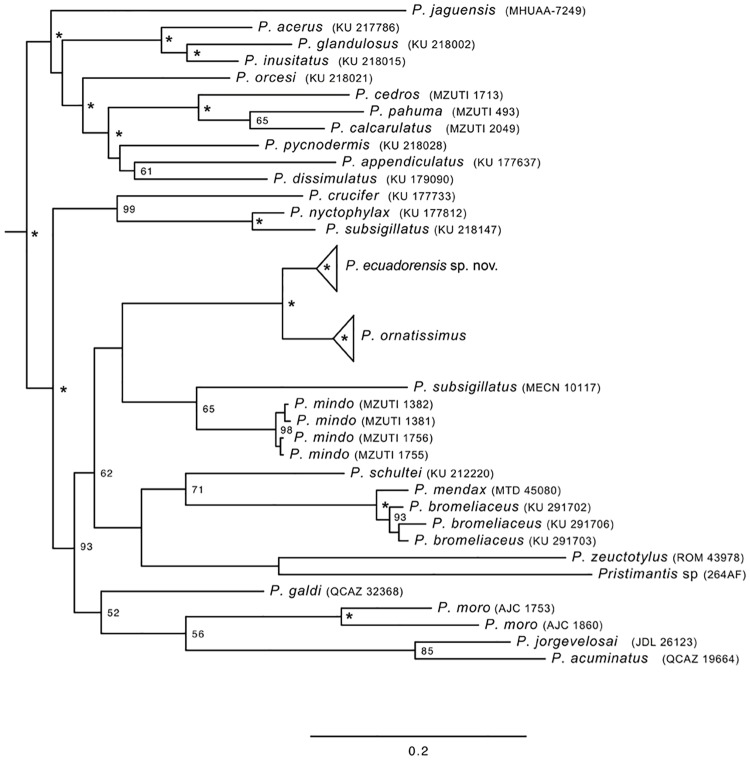
Trimmed phylogeny of *Pristimantis* showing only the most closely related species to *P*. *ornatissimus* and *P*. *ecuadorensis* sp. nov. The full phylogeny is shown as a supplementary figure (S1 Fig). Support values are presented as bootstraps and posterior probabilities.

S1 Fig provides an up-to-date hypothesis of evolutionary relationships within *Pristimantis* based on our three mtDNA gene sequences. The sister relationship of *P*. *ornatissimus* sensu stricto and the new species (described below) is well supported (bootstrap = 100, posterior probability = 1; Fig 6); their closest relatives are species found in several ecoregions, including the Andes (*P*. *mindo*, *P*. *shultei*, *P*. *bromeliaceus*, *P*. *mendax*, *P*. *nyctophylax*), lowland forest of the Chocó (*P*. *subsigillatus*), or Amazonian lowlands (*P*. *zeuctotylus*).

### Systematics

***Pristimantis ecuadorensis* sp. nov.** Guayasamin, Hutter, Tapia, Culebras, Peñafiel, Pyron, Morochz, Funk, Arteaga.

urn:lsid:zoobank.org:act:57C5D885-D927-471B-A23D-D3E555834EF1

*Eleutherodactylus ornatissimus* Lynch & Duellman 1997 [15], in part.

*Pristimantis ornatissimus* Arteaga, Bustamante & Guayasamin 2013 [20], in part.

**Suggested common name in English:** Ecuadorian rainfrog

**Suggested common name in Spanish:** Cutín de Ecuador

**Fig 7:**
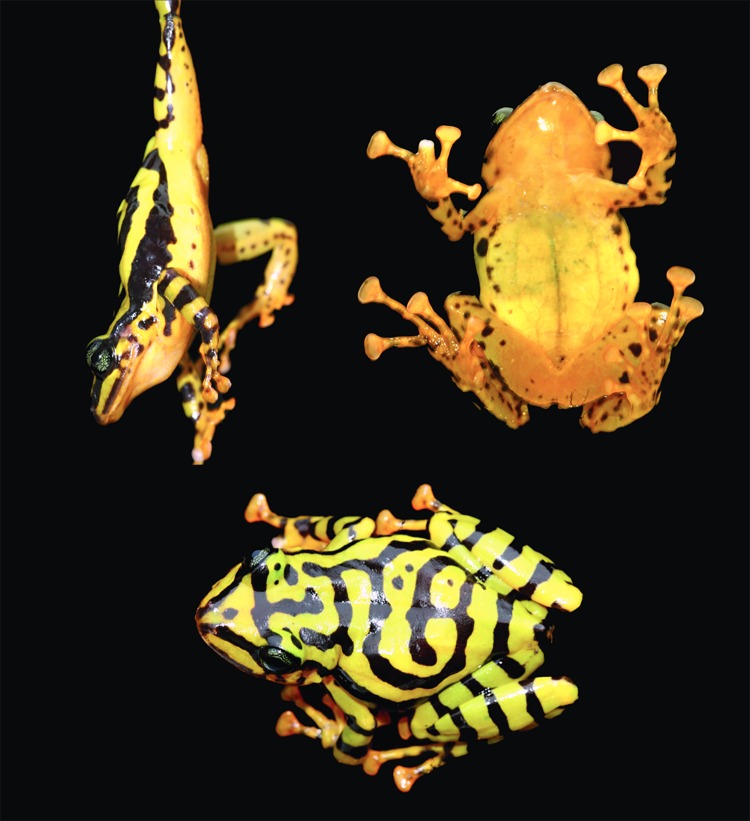
Holotype of *Pristimantis ecuadorensis* sp. nov., CJ 4084, adult female.

**Holotype:** CJ 4084 (Fig 7), adult female from a farm (0.424° S, 78.957° W; 1480 m) owned by César Tapia, ca. 3 km NE of San Francisco de Las Pampas, Cotopaxi province, Ecuador, collected on January 14^th^, 2014, by Elicio E. Tapia. Genbank accession numbers: KU574586, KU574601, KU720467.

**Paratopotypes:** CJ 4060, juvenile. CJ 5350, adult female. CJ 5351, adult male. Same data as holotype.

**Paratypes:** KU 221684, juvenile female from Palo Quemado (0.43° S, 78.91° W; 1467 m), Cotopaxi province, Ecuador, collected on 1988 by Giovanni Onore.

**Referred specimens:** MHNG 2251.090–91, 2271.097–98, 2213.083–84, from San Francisco de Las Pampas, Cotopaxi province, Ecuador; MHNG 2251.061–65, from Tandapi, Pichincha province, Ecuador; MHNG 2571.075, from Palo Quemado, Cotopaxi province, Ecuador; all collected by Giovanni Onore. These specimens are not assigned as paratypes since locality information is not precise and refer to several localities near San Francisco de Las Pampas.

CJ 4082, 4083 from a farm (0.424° S, 78.957° W; 1480 m) owned by César Tapia, ca. 3 km NE of San Francisco de Las Pampas, Cotopaxi province, Ecuador, maintained alive in situ, for conservation proposes, by the Centro Jambatu de Investigación y Conservación de Anfibios. These specimens are not assigned as paratypes since the individuals are presently alive and have not formally been deposited into a scientific collection.

**Diagnosis:** *Pristimantis ecuadorensis* is characterized by the following combination of characters: (1) skin on dorsum shagreen, that on venter smooth; discoidal fold defined posteriorly, (2) tympanic membrane and tympanic annulus evident, oval, (3) snout long, acuminate in dorsal view, rounded in profile, (4) upper eyelid lacking tubercles, (5) dentigerous process of the vomer present, bearing teeth, (6) males having vocal slits and Type I nuptial pads, (7) first finger shorter than second, (8) fingers with lateral fringes, (9) ulnar tubercles absent, (10) heel and tarsus lacking tubercles or folds, (11) inner metatarsal tubercle oval, 4–5x round outer metatarsal tubercle, (12) toes bearing lateral fringes; webbing absent; discs large; fifth toe much longer than third, (13) in life, greenish yellow dorsum with transversal black stripes that may form a reticulated pattern; iris light blue to grayish green or grayish yellow; in preservative, dorsum cream with black stripes, and (14) SVL in adult males 25.4 mm (*n* = 1) and 37.1–40.2 in adult females (*n* = 2).

**Comparison with similar species:** *Pristimantis ecuadorensis* is one of the most colorful species in the genus *Pristimantis*. It is easily distinguished from most of its congenerics by having a yellow dorsum with black stripes. Only its sister species, *P*. *ornatissimus*, has a similar pattern. The two species can be distinguished by their dorsal color pattern; *P*. *ornatissimus* has black longitudinal stripes, blotches or spots, whereas *P*. *ecuadorensis* has transverse black stripes. Also, the iris of *P*. *ornatissimus* is yellow, but light blue to grayish green or grayish yellow in *P*. *ecuadorensis*. Finally, although both species are endemic to northwestern Ecuador, they have allopatric distributions, with *P*. *ornatissimus* being restricted to elevations below 1100 m, whereas *P*. *ecuadorensis* is found at higher elevations (1467–1480 m).

**Description of holotype:** Adult female, with relatively robust body (Fig 7). Skin of dorsum and venter smooth. Head slightly longer than wide (Head Length = 38.6% of SVL; Head Width = 37.3% of SVL). Snout rounded acuminate in dorsal view and rounded in profile; *canthus rostralis* distinct, slightly concave; lips rounded, not flared. Black canthal stripe present. Nostrils slightly protuberant, directed anterolaterally. Internarial region and top of head flat. Interorbital distance longer than upper eyelid. Eye prominent, its diameter about 10.9% of SVL. Tympanic membrane clearly defined; tympanum conspicuous, oval, its diameter about 4.7% of SVL. Supratympanic fold developed, starting at posterior end of upper eyelid and reaching posterior margin of insertion of arm; black supratympanic stripe present. Dentigerous processes of vomers conspicuous, slightly curved, well-separated from each other; each process bears 10 (right) and 9 (left) teeth. Choanae large, elliptical, not concealed by palatal shelf of maxillary arch. Tongue cordiform, attached overall (narrowly free around lateral and posterior margin). Forearm lacking ulnar tubercles. Fingers relatively slender, bearing small, ovoid discs; each disc expanded laterally, and with clearly defined circumferential groove; disc on Finger III wider than tympanum diameter. Relative lengths of fingers I < II < IV < III. Fingers with dermal fringes; webbing absent. Subarticular tubercles round, simple, moderate-sized. Supernumerary tubercles present, fleshy and small. Palmar tubercle well-differentiated, bifid distally. Inner metacarpal tubercle large, elliptical.

Hind limbs moderately robust; tibia length 47.3% SVL; foot length 46.0% SVL. Calcar and tarsal tubercles absent. Inner metatarsal tubercle ovoid, 3–4 times size of round outer metatarsal tubercle; planar surface with small supernumerary tubercles; subarticular tubercles single, round, moderate-sized. Toes with lateral fringes; toe discs expanded into pads that bear a clearly defined circumferential groove. Relative length of toes I < II < III < V < IV; toe webbing absent.

**Color in life:** Dorsum greenish yellow with transverse black stripes that may form a reticulated pattern; black canthal and interorbital stripes; canthal stripe continuing posterior to eye as a flank stripe. Forearms and legs greenish yellow with black bars; venter uniform yellow. Iris light blue to grayish green or grayish yellow (Fig 2).

**Color in preservative:** Dorsum cream with black transverse stripes that may for a reticulated pattern; venter cream.

**Measurements of the holotype (in mm):** CJ 4084, adult female. SVL 40.2; Femur length 17.8; Tibia length 19.0; Foot length 18.5; Head length 15.5; Head width 15.0; Snout-to-eye distance 6.9; Tympanum 1.9; Radioulna length 8.6; Hand length 12.5; Eye diameter 4.4; Interorbital distance 5.4; Finger I length 7.7; Finger II length 9.2; Finger III Disc Diameter 3.6; Toe IV length 13.4; Toe V length 15.0; Toe IV Disc Diameter 3.4.

**Variation and sexual dimorphism:** Females are larger than males (adult male SVL 25.4–27.4 mm, *n* = 2; adult female SVL 37.1–40.5 mm, *n* = 4). Males have Type I nuptial pads (sensu Flores, 1985) and conspicuous vocal slits.

**Distribution:** *Pristimantis ecuadorensis* is known only from three nearby localities on the western slopes of the Ecuadorian Andes, provinces of Cotopaxi and Pichincha, at elevations between 1450–1480 m. The localities are: 3 km NE of San Francisco de Las Pampas (0.424° S, 78.957° W; 1480 m; Fig 1), Palo Quemado (0.43° S, 78.91° W; 1467 m), and Tandapi (0.41° S, 78.79° W; 1450 m). With the information at hand, the distribution of *P*. *ornatissimus* sensu stricto is constrained to the Chocoan lowlands and Pacific Andean foothills (< 1100 m) of Ecuador. See [15], and Fig 1.

**Fig 8:**
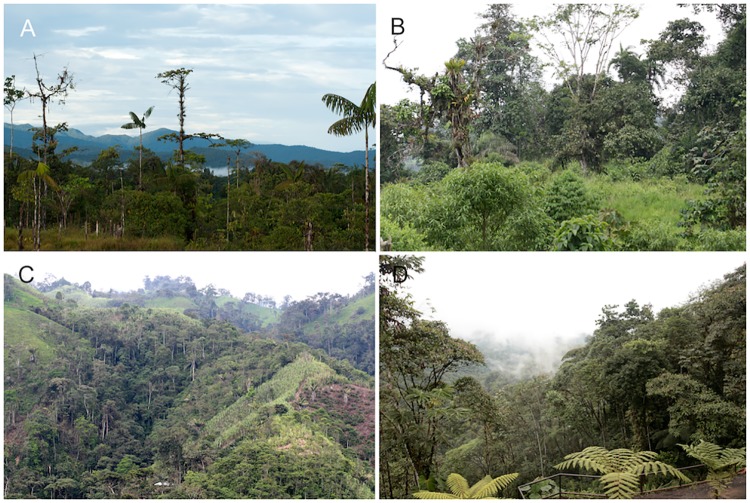
Habitat of *Pristimantis ornatissimus* sensu stricto (A, B, D) and *P*. *ecuadorensis* sp. nov (C).

**Fig 9:**
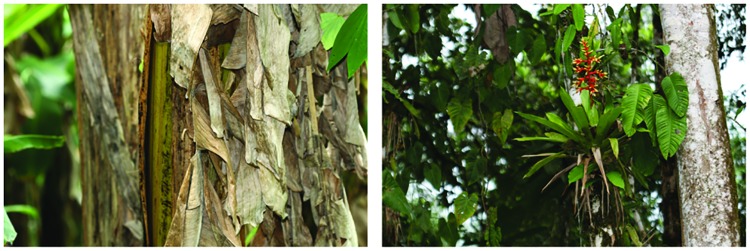
Microhabitat of *Pristimantis ecuadorensis* sp. nov. and *P*. *ornatissimus* sensu stricto.

**Natural History:** Information for *Pristimantis ecuadorensis* is mainly available from the type locality (Fig 8), San Francisco de Las Pampas, a forested valley along the Río Toachi, located at 1480 m in the northwestern slopes of the Ecuadorian Andes. The locality has a mean annual precipitation of 2325 mm (http://es.climate-data.org/location/180958/). In this area, *P*. *ecuadorensis* is found in primary forest and closely associated with the leaf axils of bromeliads, *Heliconia* plants and palm fronds (genera *Ceroxylon* and *Wettinia*) (field notes of Giovanni Onore, César Tapia, and W. E. Duellman). Additionally, the species is associated with banana (*Musa paradisiaca*) and sugar cane plantations (*Saccharum officinarum*) bordering native forest (Fig 9). In these ecosystems, *P*. *ecuadorensis* perch on top of leaves or inside leaf axils (~15–150 cm above the ground), creased leaves or moss of epiphytic plants, and have been heard calling from them. Additionally, by inspecting fecal samples, we found the remains of beetles, crickets and spiders. In captivity, females of *P*. *ecuadorensis* reach sexual maturity after 14 months, and males start vocalizing after 10 months.

**Conservation:** *Pristimantis ecuadorensis* is restricted to a very small area in the Cotopaxi and Pichincha provinces (Fig 1). In 1987, at the type locality, *P*. *ecuadorensis* was considered to be common (field notes of Giovanni Onore), as well as other syntopic amphibians now believed to be extinct (e.g., *Atelopus longirostris* and *Rhaebo caeruleostictus*). Collections housed at MNHG (Cotopaxi province: 72 specimens from San Francisco de Las Pampas, 29 specimens from Tandapi, 1 from Palo Quemado, 2 from Galápagos) corroborate that *P*. *ecuadorensis* was a relatively abundant species in the 1980s. However, our recent surveys suggest *P*. *ecuadorensis* is currently rare (i.e., only found after extensive search efforts; EET, pers. obs.). Since 2012, we have found *P*. *ecuadorensis* only at the type locality, despite surveying neighboring river valleys at the same elevation. Additionally, at San Francisco de Las Pampas, most native vegetation cover has been replaced by exotic grass for cattle grazing, and crops of banana, sugar cane, naranjilla, and tomato. Following IUCN [43] guidelines, we consider *P*. *ecuadorensis* as Endangered (Criteria A2a,c, B2a,biii).

**Etymology:** The specific name *ecuadorensis* refers to the Republic of Ecuador, where the species is endemic. The name is intended to highlight the overwhelming beauty, and cultural and biological diversity of Ecuador.

## Discussion

### Systematics

Species delimitation is a contentious issue, especially in groups were intraspecific variation is high [15, 20, 44, 45]. Herein we show how morphological and genetic data are congruent and support the recognition of a new species in what was formerly known as *Pristimantis ornatissimus*. Even though molecular differentiation between *P*. *ornatissimus* sensus stricto and *P*. *ecuadorensis* is relatively high (2.7% in 12S, 5.7% in 16S, and 9.6% in ND1; Fig 4), we stress that species delimitation cannot rely only on genetic thresholds [46], because the magnitude of intraspecific divergence can vary greatly from lineage to lineage [47]. Similarly, in groups where color polymorphism is high (e.g. *Pristimantis* [15,20]; Fig 1), taxonomic decisions should not be based only on color differences. *Pristimantis ornatissimus* sensu lato is a very good example where distinctive color patterns easily emerge among geographically separated populations; thus, taxonomic revisions necessarily require the use of additional sets of data. We highlight the importance of congruence between datasets in taxonomy because the accumulation of differences is an expected result of independent lineage evolution [48].

### Diversification

In amphibians, most studies have shown that sister species inhabit similar environments, suggesting that diversification is mostly through allopatric speciation combined with niche conservatism [8,9,11,49,50,51,52]. Our study illustrates how diversification and speciation can have multiple simultaneous patterns in a relatively small, but complex, topographic landscape. We show, for example, how color patterns can be delimited by geographic barriers (i.e. rivers, elevational gradients), which have also been suggested in other amphibians [52,53,54,55]. Additionally, we show that genetic differentiation between sister species (*P*. *ornatissimus* and *P*. *ecuadorensis*) is not explained by models of isolation by distance nor isolation by environment (Table 2).

The importance of elevational gradients in the diversification of amphibians was first suggested 20 years ago [15]; however, only recently has evidence been provided that speciation can occur across elevational gradients in the Andes [11,56]. Similar cases have also been found in reptiles [57]. Sister species found as elevational replacements of each are used as examples of the capacity of adaptation into new habitats. However, our study shows that differences in environment do not explain the genetic variation found between analyzed populations (Table 2). Instead, it is possible that speciation resulted from a geographic barrier or stochastic historic extinction of connecting populations, allowing the separate evolution of sister lineages [48]. Still, we cannot rule out the presence of environmental differences that are not captured by the WorldClim dataset; thus, analyses with variables at finer scales are needed [58]. The speciation pattern in our study system is best explained by peripatric speciation [59,60]. From an ancestral species a dispersal event produces an isolated population that evolves independently, leading to the evolution of reproductive isolation and eventual speciation. If secondary contact occurs, species are likely to evolve prezygotic isolation (e.g., call divergence, as suggested in glassfrogs and other rainfrogs; [61,62]).

We also note the importance of geography for generating genetic structure and morphological differences (Figs 1, 4 and 5). Distinctive dorsal color patterns of *Pristimantis ornatissimus* sensu stricto (Fig 1) are geographically limited (e.g., Guayllabamba-Esmeraldas river), suggesting that limited gene flow maintains these observed differences, supporting the idea that rivers can act as a barrier in terrestrial anurans [52,54,55]. Since *Pristimantis* species reproduce during the night, we do not expect a component of sexual selection in the generation and maintenance of the distinctive color patterns.

The results of this study suggest that topographically complex environments facilitate diversification in anurans. This is particularly true for *Pristimantis*, a genus with ~500 recognized species [24], where phylogeographic studies usually find marked genetic structure [56,63,64,65]. Two main factors seem to explain the extremely high species richness in *Pristimantis*, a high mutation rate [66] and low vagility [67]. Low vagility can be influenced by several variables, including size (i.e., dispersal distance increases with body size; [50,67]), physiological breadth [68,69], and reproductive mode (i.e., terrestrial).

We speculate that the role of terrestrial reproduction on anuran diversification may be related to a species’ dispersal ability in a given landscape. Amphibians with biphasic life cycles disperse using both land and water [70]; most importantly, tadpoles may move relatively long distances through streams and rivers in relatively short periods of times because of active or passive movement (e.g., flash floods). In contrast, terrestrial species only disperse through land, where habitat heterogeneity inhibits dispersal across the landscape producing isolation and metapopulation structure [71,72,73,74]. Additionally, philopatry in anurans with terrestrial reproduction could cause high genetic structure [48], a pattern that would be less pronounced in species with stream tadpoles, which generally lack marked philopatry [75]. If this general scenario is true, we would predict lower genetic differentiation within anurans that have stream tadpoles compared to terrestrial breeders. This idea is supported by Paz et al. [52] in an extensive comparative phylogeographic study that included ecological and life-history variables, as well as genetic information of 31 anuran species. Additionally, a recent study on Amazonian frogs [55] also found that species lacking tadpoles exhibited genetic structure associated with rivers as barriers, a scenario congruent with our results.

In the Andes, we expect that a myriad of new species will be discovered as phylogeographic studies become more common. We can only hope that the new discoveries are coupled with taxonomic descriptions and conservation actions, taking into account not only their distribution, but also specific threats and ecological requirements [76,77].

## Supporting information

S1 Fig Bayesian and maximum likelihood phylogenies of *Pristimantis*. Support values are presented as bootstraps and posterior probabilities.
(TIFF)

S1 Table The loadings from the three PC axes used for the MMRR analysis. (DOCX)

S2 Table Results of the MMRR analysis. The analysis uses species’ genetic distances and tests whether geographic and environmental dissimilarity influence genetic differentiation among populations. This alternative analysis uses the raw climatic variables rather than the axes from a PCA analysis. We assessed the 19 bioclimatic variables for multicolinearity by constructing a Pearson-product correlation matrix from the climatic data. Each variable selected represents one variable from a group of strongly correlated variables (using an arbitrary *p* > 0.75 as the threshold). The results are consistent with those using the first three axes from the PCA and were not significant.
(DOCX)

S1 Appendix List of species and corresponding GenBank numbers used to infer the *Pristimantis* phylogeny. (DOCX)
