# Supplementary figures and images for: Diversification of the rainfrog Pristimantis ornatissimus in the lowlands and Andean foothills of Ecuador
Source: PLoS One. 2017 Mar 22;12(3):e0172615. doi: 10.1371/journal.pone.0172615 (PMC5362048; doi:10.1371/journal.pone.0172615)

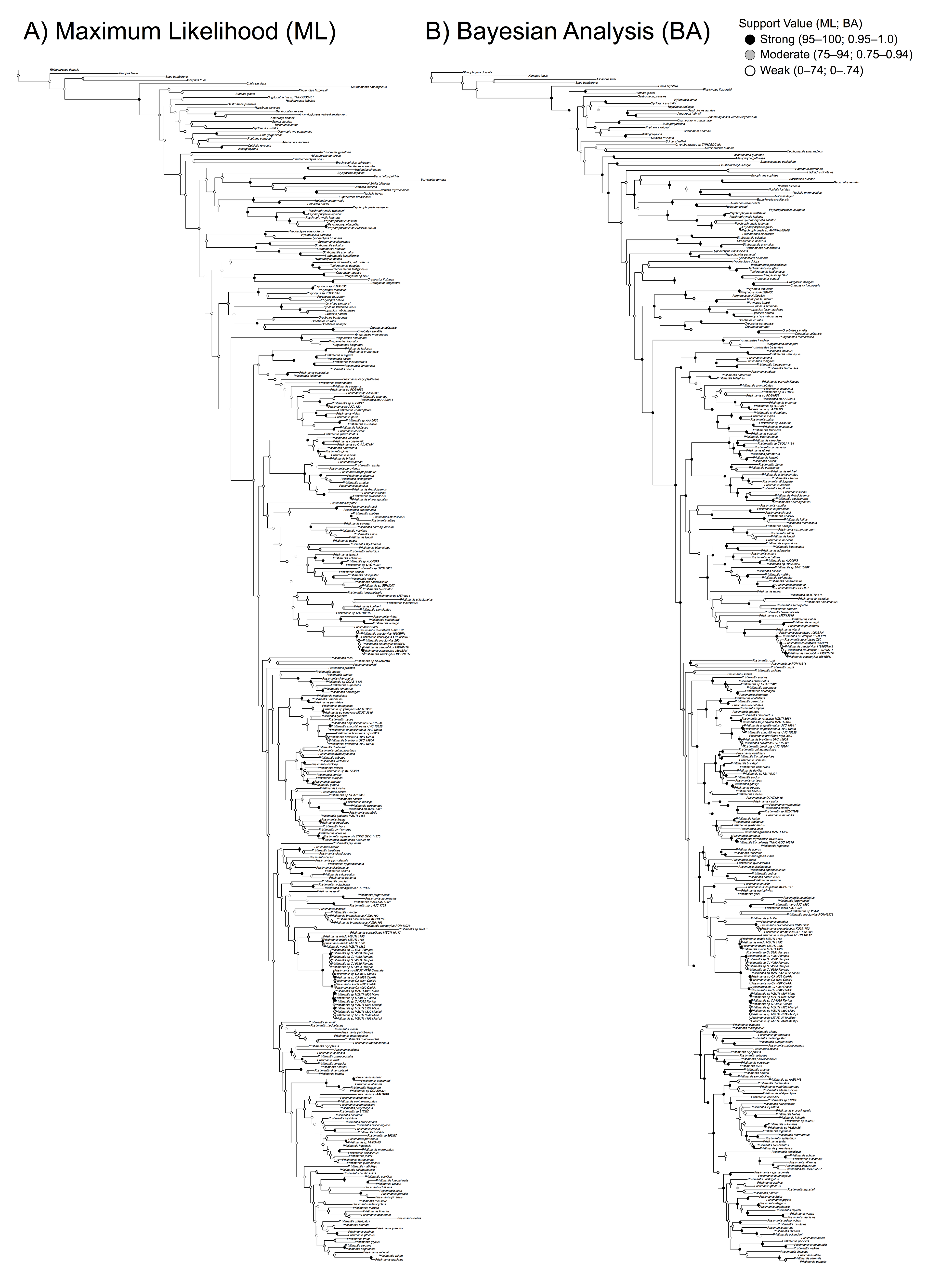

Supplement: S1 Fig — Support values are presented as bootstraps and posterior probabilities. (TIFF) [file pone.0172615.s001.tiff]
