# Supplementary material for: Diversification of the rainfrog Pristimantis ornatissimus in the lowlands and Andean foothills of Ecuador
Source: PLoS One. 2017 Mar 22;12(3):e0172615. doi: 10.1371/journal.pone.0172615 (PMC5362048; doi:10.1371/journal.pone.0172615)
Supplement: S1 Table — (DOCX) [file pone.0172615.s002.docx]

**S1 Table.** The loadings from the three PC axes used for the MMRR analysis.

|  | **PC1** | **PC2** | **PC3** |
| --- | --- | --- | --- |
| BIO1 = Annual Mean Temperature | 0.284 | -0.036 | 0.205 |
| BIO2 = Mean Diurnal Range  (Mean of monthly (max temp - min temp)) | -0.253 | 0.09 | 0.224 |
| BIO3 = Isothermality (BIO2/BIO7) (* 100) | 0.023 | 0.422 | -0.054 |
| BIO4 = Temperature Seasonality (standard deviation *100) | 0.167 | -0.339 | 0.189 |
| BIO5 = Max Temperature of Warmest Month | 0.264 | -0.067 | 0.307 |
| BIO6 = Min Temperature of Coldest Month | 0.291 | -0.008 | 0.149 |
| BIO7 = Temperature Annual Range (BIO5-BIO6) | -0.247 | -0.124 | 0.247 |
| BIO8 = Mean Temperature of Wettest Quarter | 0.281 | -0.063 | 0.211 |
| BIO9 = Mean Temperature of Driest Quarter | 0.287 | 0 | 0.19 |
| BIO10 = Mean Temperature of Warmest Quarter | 0.282 | -0.065 | 0.206 |
| BIO11 = Mean Temperature of Coldest Quarter | 0.285 | -0.011 | 0.206 |
| BIO12 = Annual Precipitation | 0.251 | 0.129 | -0.315 |
| BIO13 = Precipitation of Wettest Month | 0.182 | -0.242 | -0.384 |
| BIO14 = Precipitation of Driest Month | 0.179 | 0.329 | -0.165 |
| BIO15 = Precipitation Seasonality (Coefficient of Variation) | -0.073 | -0.414 | -0.094 |
| BIO16 = Precipitation of Wettest Quarter | 0.193 | -0.238 | -0.375 |
| BIO17 = Precipitation of Driest Quarter | 0.179 | 0.336 | -0.115 |
| BIO18 = Precipitation of Warmest Quarter | 0.226 | -0.199 | -0.297 |
| BIO19 = Precipitation of Coldest Quarter | 0.189 | 0.325 | 0.027 |
