# Supplementary material for: Diversification of the rainfrog Pristimantis ornatissimus in the lowlands and Andean foothills of Ecuador
Source: PLoS One. 2017 Mar 22;12(3):e0172615. doi: 10.1371/journal.pone.0172615 (PMC5362048; doi:10.1371/journal.pone.0172615)
Supplement: S2 Table — The analysis uses species’ genetic distances and tests whether geographic and environmental dissimilarity influence genetic differentiation among populations. This alternative analysis uses the raw climatic variables rather than the axes from a PCA analysis. We assessed the 19 bioclimatic variables for multicolinearity by constructing a Pearson-product correlation matrix from the climatic data. Each variable selected represents one variable from a group of strongly correlated variables (using an arbitrary p > 0.75 as the threshold). The results are consistent with those using the first three axes from the PCA and were not significant. (DOCX) [file pone.0172615.s003.docx]

**S2 Table.** The results of the MMRR analysis, which used species’ genetic distances and tests whether geographic and environmental dissimilarity influence genetic differentiation among populations. This alternative analysis uses the raw climatic variables rather than the axes from a PCA analysis. We assessed the 19 bioclimatic variables for multicolinearity by constructing a Pearson-product correlation matrix from the climatic data. Each variable selected represents one variable from a group of strongly correlated variables (using an arbitrary *r* > 0.75 as the threshold). The results are consistent with those using the first three axes from the PCA and were not significant.

| **Variable** | **Coefficient** | **T statistic** | **T P-value** | **F statistic** | **F p-value** |
| --- | --- | --- | --- | --- | --- |
| BIO13 | 0 | 1.605 | 0.112 | 0.973 | 0.435 |
| BIO4 | 0 | -0.798 | 0.411 |  |  |
| Geographic | 0 | -0.571 | 0.509 |  |  |
| BIO15 | 0 | 0.362 | 0.705 |  |  |
| Intercept | 0.031 | 7.561 | 0.707 |  |  |
| BIO12 | 0 | 0.215 | 0.838 |  |  |
| BIO1 | 0 | 0 | 1 |  |  |
