# Supplementary material for: Diversification of the rainfrog Pristimantis ornatissimus in the lowlands and Andean foothills of Ecuador
Source: PLoS One. 2017 Mar 22;12(3):e0172615. doi: 10.1371/journal.pone.0172615 (PMC5362048; doi:10.1371/journal.pone.0172615)
Supplement: S1 Appendix — (DOCX) [file pone.0172615.s004.docx]

**S1 Appendix.** List of species and GenBank codes used to infer the *Pristimantis* phylogeny.

| **Family and species** | **GenBank code** | | |
| --- | --- | --- | --- |
|  | **12S** | **16S** | **ND1** |
| **Ascaphidae** |  |  |  |
| *Ascaphus truei* | - | - | AJ871087 |
| **Brachycephalidae** |  |  |  |
| *Brachycephalus ephippium* | AY326008 | AY326008 | GQ345243 |
| *Ischnocnema guentheri* | EF493533 | EF493533 | - |
| **Bufonidae** |  |  |  |
| *Bufo gargarizans* | DQ275350 | FJ882843 | NC_008410 |
| *Osornophryne guacamayo* | AY819334 | AY326036 | AY819464 |
| **Centrolenidae** |  |  |  |
| *Celsiella revocata* | EU663379 | EU663019 | EU663113 |
| *Ikakogi tayrona* | EU663356 | EU662997 | EU663091 |
| *Ceuthomantis smaragdinus* | GQ345132 | GQ345133 | GQ345251 |
| **Craugastoridae** |  |  |  |
| *Barycholos pulcher* | EU186727 | EU186709 | - |
| *Barycholos ternetzi* | - | DQ283094 | - |
| *Bryophryne cophites* | EF493537 | EF493537 | - |
| *Craugastor augusti* | JX564870 | JX564870 | JX564870 |
| *Craugastor fitzingeri* | AY326001 | AY326001 | GQ345245 |
| *Craugastor longirostris* | EF493395 | EF493395 | - |
| *Craugastor sp UAZ* | DQ283271 | DQ283271 | - |
| *Euparkerella brasiliensis* | JX298276 | JX267468 | - |
| *Haddadus aramunha* | KF740844 | KF740845 | - |
| *Haddadus binotatus* | EF493361 | EF493361 | - |
| *Holoaden bradei* | EF493378 | EF493366 | - |
| *Holoaden luederwaldti* | EU186728 | EU186710 | - |
| *Hypodactylus brunneus* | EF493357 | EF493357 | GQ345248 |
| *Hypodactylus dolops* | EF493394 | EF493394 | - |
| *Hypodactylus elassodiscus* | EF493358 | EF493358 | - |
| *Hypodactylus peraccai* | EF493710 | EF493710 | - |
| *Lynchius flavomaculatus* | EU186667 | EU186667 | - |
| *Lynchius nebulanastes* | EU186704 | EU186704 | - |
| *Lynchius parkeri* | EU186705 | EU186705 | - |
| *Lynchius simmonsi* | JF809940 | JF810004 | - |
| *Noblella bilineata* | JX267393 | JX267323 | - |
| *Noblella heyeri* | JX267463 | JX267541 | - |
| *Noblella lochites* | EU186699 | EU186699 | - |
| *Noblella myrmecoides* | JX267464 | JX267542 | - |
| *Oreobates barituensis* | JF809935 | JF809999 | - |
| *Oreobates cruralis* | EU186666 | EU186666 | - |
| *Oreobates pereger* | JF809926 | JF809956 | - |
| *Oreobates quixensis* | AY819344 | JF810003 | AY819474 |
| *Oreobates saxatilis* | DQ283060 | DQ283061 | - |
| *Phrynopus bracki* | EF493709 | EF493709 | - |
| *Phrynopus sp* KU291630 | EU186725 | EU186707 | - |
| *Phrynopus sp* KU291634 | EF493708 | EF493708 | - |
| *Phrynopus tautzorum* | AM039720 | AM039652 | - |
| *Phrynopus tribulosus* | EU186725 | EU186707 | - |
| *Pristimantis acatallelus* | - | JN104675 | - |
| *Pristimantis acerus* | EF493678 | EF493678 | - |
| *Pristimantis achatinus* | EF493827 | EF493660 | - |
| *Pristimantis achuar* | - | EU130626 | - |
| *Pristimantis actites* | EF493696 | EF493696 | - |
| *Pristimantis acuminatus* | - | EU130579 | - |
| *Pristimantis adiastolus* | AY964086 | - | - |
| *Pristimantis affinis* | JN991487 | JN991424 | - |
| *Pristimantis albertus* | EU186695 | - | - |
| *Pristimantis altae* | JN991496 | - | - |
| *Pristimantis altamazonicus* | EF493670 | EF493670 | - |
| *Pristimantis altamnis* | - | EU130617 | - |
| *Pristimantis aniptopalmatus* | EU186694 | EU186694 | - |
| *Pristimantis anolirex* | - | DQ195450 | - |
| *Pristimantis appendiculatus* | EF493524 | EF493524 | - |
| *Pristimantis ardalonychus* | EU186664 | EU186664 | - |
| *Pristimantis aureoventris* | - | JQ742151 | JQ742333 |
| *Pristimantis bambu* | JF906319 | - | - |
| *Pristimantis bipunctatus* | EF493702 | EF493702 | - |
| *Pristimantis bogotensis* | JN991497 | DQ195451 | - |
| *Pristimantis boulengeri* | - | DQ195452 | - |
| *Pristimantis briceni* | JX155297 | JX155297 | - |
| *Pristimantis buccinator* | - | EU712630 | - |
| *Pristimantis buckleyi* | EF493350 | EF493350 | - |
| *Pristimantis cajamarcensis* | EF493823 | EF493663 | - |
| *Pristimantis calcaratus* | - | JN104657 | - |
| *Pristimantis calcarulatus* | - | EF493523 | - |
| *Pristimantis caprifer* | EF493391 | EF493391 | - |
| *Pristimantis carranguerorum* | - | DQ195453 | - |
| *Pristimantis carvalhoi* | - | DQ195454 | - |
| *Pristimantis caryophyllaceus* | EU186686 | EU186686 | - |
| *Pristimantis cedros* | - | EF493523 | - |
| *Pristimantis celator* | EF493685 | EF493685 | - |
| *Pristimantis cerasinus* | JN991502 | FJ784387 | - |
| *Pristimantis ceuthospilus* | EF493520 | EF493520 | - |
| *Pristimantis chalceus* | EF493675 | EF493675 | - |
| *Pristimantis chiastonotus* | - | JN691273 | - |
| *Pristimantis chloronotus* | AY326007 | AY326007 | - |
| *Pristimantis citriogaster* | EF493700 | EF493700 | - |
| *Pristimantis colomai* | EF493354 | EF493354 | - |
| *Pristimantis condor* | EF493701 | EF493701 | - |
| *Pristimantis conservatio* | JX155287 | JX155281 | - |
| *Pristimantis conspicillatus* | EF493529 | EF493529 | - |
| *Pristimantis cremnobates* | EF493528 | EF493528 | - |
| *Pristimantis crenunguis* | EF493693 | EF493666 | - |
| *Pristimantis croceoinguinis* | EF493669 | EF493665 | - |
| *Pristimantis crucifer* | EU186736 | EU186718 | - |
| *Pristimantis cruciocularis* | EU186656 | EU186656 | - |
| *Pristimantis cruentus* | EF493697 | EF493697 | FJ882747 |
| *Pristimantis cryophilius* | EF493672 | EF493672 | - |
| *Pristimantis curtipes* | EF493513 | EF493513 | AY819473 |
| *Pristimantis danae* | - | EU192266 | - |
| *Pristimantis devillei* | EF493688 | EF493688 | - |
| *Pristimantis diadematus* | EU186668 | EU186668 | - |
| *Pristimantis dissimulatus* | EF493522 | EF493522 | - |
| *Pristimantis duellmani* | AY326003 | AY326003 | - |
| *Pristimantis elegans* | - | DQ195457 | - |
| *Pristimantis eriphus* | EU186671 | EU186671 | - |
| *Pristimantis erythropleura* | JN991509 | JN371036 | - |
| *Pristimantis euphronides* | EF493527 | EF493527 | - |
| *Pristimantis fenestratus* | FJ438809 | EF493703 | - |
| *Pristimantis festae* | EF493515 | EF493515 | - |
| *Pristimantis frater* | - | DQ195459 | - |
| *Pristimantis gaigei* | JN991511 | JN991449 | - |
| *Pristimantis galdi* | EU186670 | EU186670 | - |
| *Pristimantis gentryi* | EF493511 | EF493511 | - |
| *Pristimantis ginesi* | JX155295 | JX155295 | - |
| *Pristimantis glandulosus* | EF493676 | EF493676 | - |
| *Pristimantis gryllus* | JX306022 | JX306022 | - |
| *Pristimantis hectus* | - | JN104680 | - |
| *Pristimantis imitatrix* | EF493824 | EF493667 | - |
| *Pristimantis inguinalis* | EU186676 | EU186676 | - |
| *Pristimantis inusitatus* | EF493677 | EF493677 | - |
| *Pristimantis jester* | - | JQ742169 | JQ742341 |
| *Pristimantis jorgevelosai* | - | DQ195461 | - |
| *Pristimantis juanchoi* | - | JN104681 | - |
| *Pristimantis jubatus* | - | JN104663 | - |
| *Pristimantis kelephas* | - | JN104660 | - |
| *Pristimantis kichwarum* | - | EU130582 | - |
| *Pristimantis koehleri* | FJ438810 | FJ438799 | - |
| *Pristimantis labiosus* | EF493694 | EF493694 | - |
| *Pristimantis lancinii* | JX155294 | JX155283 | - |
| *Pristimantis lanthanites* | EF493695 | EF493695 | - |
| *Pristimantis latidiscus* | EF493698 | EF493698 | - |
| *Pristimantis leoni* | EF493684 | EF493684 | - |
| *Pristimantis librarius* | JN991515 | JN991451 | - |
| *Pristimantis lirellus* | EF493521 | EF493521 | - |
| *Pristimantis llojsintuta* | - | EU712641 | - |
| *Pristimantis luteolateralis* | EF493517 | EF493517 | - |
| *Pristimantis lutitus* | - | DQ195464 | - |
| *Pristimantis lymani* | EF493392 | EF493392 | - |
| *Pristimantis lynchi* | - | DQ195463 | - |
| *Pristimantis malkini* | EU186663 | EU186663 | - |
| *Pristimantis marmoratus* | EU186692 | EU201063 | - |
| *Pristimantis martiae* | JN991516 | - | - |
| *Pristimantis mashpi* | KM675441 | KM675466 | - |
| *Pristimantis melanogaster* | EF493826 | EF493664 | - |
| *Pristimantis mendax* | EU186659 | EU186659 | - |
| *Pristimantis merostictus* | - | DQ195465 | - |
| *Pristimantis minutulus* | EU186657 | EU186657 | - |
| *Pristimantis miyatai* | JN991518 | DQ195466 | - |
| *Pristimantis museosus* | JN991521 | KC014940 | - |
| *Pristimantis mutabilis* | KM675434 | KM675458 | - |
| *Pristimantis myops* | - | JN104682 | - |
| *Pristimantis nervicus* | JN991522 | JN991456 | - |
| *Pristimantis nyctophylax* | EF493526 | EF493526 | - |
| *Pristimantis ockendeni* | EF493519 | EF493519 | - |
| *Pristimantis ocreatus* | EF493682 | EF493682 | - |
| *Pristimantis orcesi* | EF493679 | EF493679 | - |
| *Pristimantis orestes* | EF493388 | EF493388 | - |
| *Pristimantis ornatus* | EU186660 | EU186660 | - |
| *Pristimantis pahuma* | - | EF493523 | - |
| *Pristimantis paisa* | JN991524 | JN991459 | - |
| *Pristimantis palmeri* | - | JN371001 | - |
| *Pristimantis paramerus* | JX155279 | JX155279 | - |
| *Pristimantis pardalis* | JN991527 | FJ784386 | - |
| *Pristimantis parvillus* | EF493352 | EF493352 | - |
| *Pristimantis paulodutrai* | JX267297 | JX267297 | - |
| *Pristimantis permixtus* | - | DQ195467 | - |
| *Pristimantis peruvianus* | - | JN991461 | - |
| *Pristimantis petrobardus* | EF493825 | EF493367 | - |
| *Pristimantis pharangobates* | AY843586 | AY843586 | - |
| *Pristimantis phoxocephalus* | EF493349 | EF493349 | - |
| *Pristimantis pirrensis* | JN991528 | JN991462 | - |
| *Pristimantis platydactylus* | FJ438811 | EU192255 | - |
| *Pristimantis pleurostriatus* | JX155292 | JX155292 | - |
| *Pristimantis pluvicanorus* | AY843586 | AY843586 | - |
| *Pristimantis prolatus* | EU186701 | EU186701 | - |
| *Pristimantis ptochus* | JN991530 | - | - |
| *Pristimantis pulvinatus* | EU186741 | EU186723 | - |
| *Pristimantis pycnodermis* | EF493680 | EF493680 | - |
| *Pristimantis pyrrhomerus* | EF493683 | EF493683 | - |
| *Pristimantis quantus* | - | JN104684 | - |
| *Pristimantis quaquaversus* | - | EU130580 | - |
| *Pristimantis quinquagesimus* | EF493690 | EF493690 | - |
| *Pristimantis ramagii* | JX267300 | JX267299 | - |
| *Pristimantis reichlei* | EF493707 | EF493707 | - |
| *Pristimantis rhabdocnemus* | EU186724 | EU186706 | - |
| *Pristimantis rhabdolaemus* | EF493706 | EF493706 | - |
| *Pristimantis rhodoplichus* | EF493674 | EF493674 | - |
| *Pristimantis ridens* | EF493355 | EF493355 | - |
| *Pristimantis riveti* | EF493348 | EF493348 | - |
| *Pristimantis rozei* | EF493691 | EF493691 | - |
| *Pristimantis sagittulus* | EF493705 | EF493705 | - |
| *Pristimantis saltissimus* | EU186693 | EU186693 | JQ742340 |
| *Pristimantis samaipatae* | FJ438814 | FJ438803 | - |
| *Pristimantis savagei* | JN991536 | DQ195470 | - |
| *Pristimantis schultei* | EF493681 | EF493681 | - |
| *Pristimantis shrevei* | EF493692 | EF493692 | - |
| *Pristimantis simonbolivari* | EF493671 | EF493671 | - |
| *Pristimantis simonsii* | EU186665 | EU186665 | - |
| *Pristimantis simoterus* | - | DQ195471 | - |
| *Pristimantis skydmainos* | EF493393 | EF493393 | - |
| *Pristimantis sobetes* | KM675428 | KM675449 | - |
| *Pristimantis* sp 264AF | JN690704 | - | - |
| *Pristimantis* sp 317MC | JN690709 | JN691316 | - |
| *Pristimantis* sp 395MC | JN690706 | JN691314 | - |
| *Pristimantis* sp AAA5635 | - | KC129357 | - |
| *Pristimantis* sp AAB3748 | - | JN991421 | - |
| *Pristimantis* sp AAB8264 | - | KC129363 | - |
| *Pristimantis* sp AJC0217 | JN991486 | KC129344 | - |
| *Pristimantis* sp AJC0573 | JN991485 | JN991420 | - |
| *Pristimantis* sp AJC1129 | - | KC129344 | - |
| *Pristimantis* sp AJC1683 | JN991489 | JN991426 | - |
| *Pristimantis* sp CVULA7184 | JX155281 | JX155281 | - |
| *Pristimantis* sp KU179221 | EU186700 | EU186700 | - |
| *Pristimantis* sp MTR13615 | JX267462 | JX267387 | - |
| *Pristimantis* sp MTR4514 | JX267461 | JX267386 | - |
| *Pristimantis* sp MZUTI909 | KM675440 | KM675463 | - |
| *Pristimantis* sp PDG1959 | JN991490 | JN991427 | - |
| *Pristimantis* sp QCAZ12410 | EF493686 | EF493686 | - |
| *Pristimantis* sp QCAZ16428 | EF493516 | EF493516 | - |
| *Pristimantis* sp QCAZ25577 | - | EU130591 | - |
| *Pristimantis* sp ROM43318 | EU186735 | EU186717 | - |
| *Pristimantis* sp SBH2007 | EF493356 | EF493356 | - |
| *Pristimantis* sp UVC15867 | - | JN104676 | - |
| *Pristimantis* sp UVC15953 | - | JN371033 | - |
| *Pristimantis* sp VUB3485 | - | JQ742166 | - |
| *Pristimantis spinosus* | EF493673 | EF493673 | - |
| *Pristimantis stictogaster* | EF493704 | EF493704 | - |
| *Pristimantis suetus* | JN991537 | JN991469 | - |
| *Pristimantis supernatis* | AY326005 | AY326005 | - |
| *Pristimantis surdus* | EF493687 | EF493687 | - |
| *Pristimantis taeniatus* | JN991539 | DQ195474 | - |
| *Pristimantis terraebolivaris* | EU186650 | EU186650 | - |
| *Pristimantis thectopternus* | - | JN104685 | - |
| *Pristimantis thymalopsoides* | EF493514 | EF493514 | - |
| *Pristimantis toftae* | EF493353 | EF493353 | - |
| *Pristimantis truebae* | EF493512 | EF493512 | - |
| *Pristimantis unistrigatus* | EF493387 | EF493387 | - |
| *Pristimantis uranobates* | - | DQ195476 | - |
| *Pristimantis urichi* | EF493699 | EF493699 | - |
| *Pristimantis vanadise* | JX155291 | JX155290 | - |
| *Pristimantis ventrimarmoratus* | JF906310 | - | - |
| *Pristimantis verecundus* | KM675424 | KM675445 | - |
| *Pristimantis versicolor* | EF493389 | EF493389 | - |
| *Pristimantis vertebralis* | EF493689 | EF493689 | - |
| *Pristimantis viejas* | JN991547 | JN991475 | - |
| *Pristimantis vinhai* | JX267343 | JX267343 | - |
| *Pristimantis walkeri* | EF493518 | EF493518 | - |
| *Pristimantis wiensi* | EF493377 | EF493668 | - |
| *Pristimantis yukpa* | JX306020 | JX306021 | - |
| *Pristimantis yuruaniensis* | - | JQ742160 | JQ742335 |
| *Pristimantis zophus* | JN991548 | JN991480 | - |
| *Psychrophrynella guillei* | AY843720 | AY843720 | - |
| *Psychrophrynella iatamasi* | - | AM039644 | - |
| *Psychrophrynella laplacai* | AM039711 | AM039643 | - |
| *Psychrophrynella saltator* | AM039710 | AM039642 | - |
| *Psychrophrynella* sp AMNHA165108 | AY843720 | AY843720 | - |
| *Psychrophrynella usurpator* | EF493714 | EF493714 | - |
| *Psychrophrynella wettsteini* | EU186696 | EU186696 | GQ345250 |
| *Strabomantis anomalus* | EF493534 | EF493534 | - |
| *Strabomantis biporcatus* | EU186691 | EU186691 | GQ345249 |
| *Strabomantis bufoniformis* | DQ283165 | DQ283165 | - |
| *Strabomantis necerus* | EF493535 | EF493535 | - |
| *Strabomantis sulcatus* | EF493536 | EF493536 | - |
| *Yunganastes ashkapara* | FJ438807 | FJ438796 | - |
| *Yunganastes bisignatus* | JF809936 | EU192234 | - |
| *Yunganastes fraudator* | - | FJ539065 | - |
| *Yunganastes mercedesae* | JF809939 | FJ539066 | - |
| **Dendrobatidae** |  |  |  |
| *Ameerega hahneli* | DQ501996 | EU342617 | HQ290998 |
| *Anomaloglossus verbeeksnyderorum* | HQ290952 | HQ290952 | HQ290952 |
| *Dendrobates auratus* | AY364565 | AY326030 | JX564862 |
| **Eleutherodactylidae** |  |  |  |
| *Adelophryne gutturosa* | EU186679 | EU186679 | GQ345247 |
| *Eleutherodactylus coqui* | GQ345176 | GQ345176 | FJ882750 |
| **Hemiphractidae** |  |  |  |
| *Cryptobatrachus* sp TNHCGDC451 | JX564861 | JX564861 | JX564861 |
| *Flectonotus fitzgeraldi* | AY819355 | DQ679381 | AY819486 |
| *Gastrotheca pseustes* | AY326051 | AY326051 | JX564866 |
| *Hemiphractus bubalus* | DQ679263 | DQ679412 | DQ679370 |
| *Stefania ginesi* | DQ679266 | JQ742173 | DQ679373 |
| **Hylidae** |  |  |  |
| *Cyclorana australis* | AY843580 | AY843580 | GQ366300 |
| *Hylomantis lemur* | AY843725 | AY843725 | GQ366294 |
| *Hypsiboas raniceps* | AY843657 | AY843657 | JQ023173 |
| *Scinax staufferi* | AY843761 | AY843761 | GQ366340 |
| **Leptodactylidae** |  |  |  |
| *Adenomera andreae* | KC520683 | KC520683 | HQ290944 |
| *Rupirana cardosoi* | KC593361 | KC603956 | KC593354 |
| **Myobatrachidae** |  |  |  |
| *Crinia signifera* | - | - | JX564860 |
| **Pipidae** |  |  |  |
| *Xenopus laevis* | - | - | HM991335 |
| **Rhinophrynidae** |  |  |  |
| *Rhinophrynus dorsalis* | - | - | NC_015620 |
| **Scaphiopodidae** |  |  |  |
| *Spea bombifrons* | - | - | JX564896 |
